# Associations between diet, exercise, and mental health among college students in Guangdong, China: A cross-sectional and interventional study

**DOI:** 10.1097/MD.0000000000047375

**Published:** 2026-01-30

**Authors:** Jia Fu, Xuelan Wu, Xi Yu, Tian Zhong, Ying Xiao, Xiaoyu Tao

**Affiliations:** a Faculty of Medicine, Macau University of Science and Technology, Taipa, Macao SAR, China; b School of Health, Zhuhai College of Science and Technology, Zhuhai, China; c School of Management, Guangzhou College of Commerce, Guangzhou, Guangdong, China; d Faculty of Innovative Hospitality Management, Macau University of Tourism, Taipa, Macao SAR, China.

**Keywords:** college students, diet balance, mental health, physical activity

## Abstract

Neglectful eating habits, a sedentary lifestyle, and psychological disturbance are common issues among college students. This study aims to identify the relationships between these factors among college students through an intervention in Guangdong, China. Data on a sample of college students (N = 683) was collected through a cross-sectional study alongside the Depression Anxiety Stress Scale-21, Diet Balance Index-16, and Physical Activity Rating Scale-3 questionnaires, all of which comprise standardized scales. An intervention trial with 440 participants (220 intervention participants and 220 control participants) was also conducted to gauge the impacts of psychological disturbances, poor diets, and a lack of physical activity. The statistical methods employed included descriptive statistics, nonparametric group comparisons, correlation analyses, and pre–post comparison. Students reported substantial stress and anxiety and exhibited depressive symptoms, with their mean scores nearing and/or crossing mild thresholds. The physical and psychosocial correlates of dietary quality were suboptimal, featuring positive associations with cereals, condiments, and energy-dense foods, and negative associations with vegetables, fruits, dairy, and protein. A majority of participants reported a low level of physical activity, though there were notable differences across genders and grade levels. Correlation analysis revealed that increases in physical activity were linked to improved mental health, while more severe dietary imbalances were associated with worsened mental health. Notably, participants in the control group exhibited greater improvements in stress, anxiety, depression, physical activity, and dietary balance compared to the intervention group (all *P* < .05). Mental health issues, low physical activity, and poor dietary patterns represent pressing concerns among college students in Guangdong. However, evidence suggests that integrated lifestyle interventions have the potential to address these issues.

## 1. Introduction

Students in college are at a very crucial point in their lives. On the verge of finishing their teenage years, they are expected to become adults. Unsurprisingly, then, they are often very susceptible to various health problems during this time. The increase in stress, anxiety, and depression levels among this demographic is generally attributed to mounting academic burdens, problems with social integration, and changes to everyday routines.^[1]^ China and several other countries have conducted comprehensive studies and observed an increase in the number of students experiencing psychological distress, particularly during and after the COVID-19 pandemic.^[2,3]^ This decline in the mental health of young adults is emerging as a serious public concern, especially given how detrimental its consequences can be to a Person academic success, social life, and overall well-being.^[4]^

Aside from psychological influences, mental health outcomes are correlated with various lifestyle choices, including nutrition and exercise. Engagement in physical activity has been shown to reduce symptoms of depression and boost psychological resilience. Cross-sectional and longitudinal studies have found that, among college students, increased physical activity is associated with reduced stress, anxiety, and depression symptoms.^[4–6]^ In contrast, sedentary activities and a lack of exercise are linked to worse mental health outcomes, underscoring the need for policies that promote physical activity in campus settings.^[2]^

The second major element is dietary quality, with poor diets (such as those featuring heavy consumption of fast food, sweetened drinks, and processed foods) associated with a greater risk of depression and anxiety.^[7,8]^ In contrast, diets that are high in fruits, vegetables, and diverse sources of proteins are linked to stronger psychological well-being.^[9,10]^ In China, for example, according to Ma et al,^[10]^ there are significant correlations between Diet Balance Index scores and indicators of physical and mental health among adolescents and young adults. While these studies underscore the link between diet and mental health, many of them analyze nutrition and physical activity in silos.

The elements of dietary quality, physical activity, and psychological health are increasingly regarded as interconnected in efforts to improve public health. Notably, youth health problems can seemingly be resolved more effectively through integrated lifestyle approaches than through single-behavior approaches.^[6,9]^ Within the context of China, very few studies have simultaneously used the Depression Anxiety Stress Scale-21 (DASS-21), the Diet Balance Index (DBI-16), and the Physical Activity Rating Scale (PARS-3) (all relevant standardized instruments) to assess these factors among college students. Additionally, intervention-based research in the country covering several lifestyle factors has been sparse, leading to a lack of evidence for any health-promotion strategies that could be reliably implemented in campus settings.

This study aims to determine the relationships among mental health, physical activity, and dietary quality among a sample of college students in the Chinese province of Guangdong. Moreover, it considers an array of implemented lifestyle changes, evaluating the specific psychological, dietary, and physical components. The results will aid in the development of integrated approaches to public health concerns among university students in China.

## 2. Methods

### 2.1. Study design and participants

This research was conducted using a sequential test model alongside a cross-sectional survey technique. For the overall sample of 683 college students in the Guangdong Province of China, a stratified cluster-sampling technique spanning 4 academic years was employed. Ultimately, 440 students volunteered to take part in the next phase and were assigned at random to the intervention group (n = 220) or the control group (n = 220). The inclusion criteria were as follows: full-time enrolled undergraduate student; between the ages of 18 and 25; and able to provide their informed consent through a signed form. Notably, participants with enduring “previously diagnosed” acute psychiatric conditions or long-term physically debilitating conditions were not included in the sample.

### 2.2. Measures

#### 2.2.1. Mental health

Mental health status was assessed using the DASS-21, which evaluates 3 distinct dimensions: depression, anxiety, and stress.^[11]^ Each item is rated along a 4-point Likert scale ranging from 0 (“Did not apply to me at all”) to 3 (“Applied to me very much, or most of the time”). The DASS-21 has been validated across a diverse range of populations (including Chinese college students) demonstrating high internal consistency and construct validity.^[12,13]^

#### 2.2.2. Dietary quality

Dietary quality was measured using the Chinese DBI-16, a validated tool that has been widely applied in nutritional epidemiology in China to assess both insufficient and excessive food consumption relative to the Dietary Guidelines for Chinese Residents.^[14]^ The DBI-16 includes sub-scores for cereals, vegetables and fruits, dairy and soy, animal-based foods, condiments, and energy-dense foods. A higher absolute diet quality distance score indicates a more substantial imbalance in dietary intake.

#### 2.2.3. Physical activity

Physical activity levels were assessed using the PARS-3, a standardized instrument commonly used to assess physical activity among Chinese populations.^[15]^ The PARS-3 calculates an overall score based on exercise intensity, frequency, and duration, with higher scores indicating higher activity levels. The scale has been widely used for public health and sports medicine research among Chinese college students.

### 2.3. Intervention procedure

The participants in the intervention group followed a 12-week lifestyle curriculum featuring the following elements: weekly workshops on healthy eating and nutrition; supervised group exercise sessions; and stress-management training, which incorporated mindfulness and relaxation techniques. The control group did not take part in any structured program and continued with their normal activities on campus. Both groups were assessed at the baseline (pretest) and after the 12-week study period (posttest) using the same instruments.

### 2.4. Statistical analysis

Descriptive statistics were computed for each primary variable, including mean, standard deviation, median, and interquartile range. Normality was tested using the Kolmogorov–Smirnov test. Differences between groups (by gender and academic year) were assessed using nonparametric methods, namely the Mann–Whitney *U* test and the Kruskal–Wallis test. The relationships between the 3 variables of interest (dietary quality, physical activity, and mental health) were assessed using Pearson correlations. The intervention’s impact was assessed through Wilcoxon signed-rank tests for within-group changes and Mann–Whitney *U* tests for between-group comparisons. A *P*-value under .05 (two-tailed) served as the cutoff for statistical significance. All statistical analysis was performed in Statistical Package for the Social Sciences 26.0 (IBM Corp., Armonk).

### 2.5. Ethics

This study has been subjected to rigorous ethical scrutiny and granted formal approval by the Institutional Review Board of Zhuhai College of Science and Technology, under the authorization code “(2024) Review No. (2024018).” Written informed consent was obtained from all participants prior to the start of data collection. All procedures were conducted in accordance with the Declaration of Helsinki.

## 3. Results

### 3.1. Participant characteristics

A total of 683 valid questionnaires were analyzed. A majority of the participants were female (61.8%), while a plurality were first-year students (34.4%), followed by sophomores (29.6%), juniors (24.3%), and seniors (11.7%) (see Table 1).

**Table 1:** Population frequency distribution.

Variable	Category	Frequency	Percentage (%)
Grade	Freshman	235	34.4
	Sophomore	202	29.6
	Junior	166	24.3
	Senior	80	11.7
Gender	Male	261	38.2
	Female	422	61.8

### 3.2. Mental health, physical activity, and dietary quality

Almost all of the participating students reported experiencing some degree of psychological distress: stress scored moderately (15.06), anxiety scored lightly (14.13), and depression scored faintly (12.71), giving way to a sum of averages across all factors of 41.9. Students engaged in very little physical activity, with the average score on the PARS-3 being just 11.98, well under the threshold of 19. Students’ average DBI-16 score of 42.69 indicates moderate dietary imbalance characterized by the excessive intake of cereals, condiments, and energy-dense foods alongside the insufficient intake of vegetables, fruits, dairy, and soy products (see Tables 2 and 3).

**Table 2:** Mental health and physical activity statistics.

Variable	N	Mean	Minimum	Maximum	Percentiles		
					*P*_25_	*P*_50_	*P*_75_
Stress	683	15.06	0	38	10	14	18
Anxiety	683	14.13	0	36	10	12	18
Depression	683	12.71	0	42	8	10	16
Overall mental health	683	41.90	0	96	28	34	46
Physical activity	683	11.98	0	40	6	9	18

*Notes*: for the depression subscale, cutoff scores of 10, 14, and 21 indicate mild, moderate, and severe depression, respectively. For the anxiety subscale, cutoff scores of 8, 10, and 15 indicate mild, moderate, and severe anxiety, respectively. For the stress subscale, cutoff scores of 15, 19, and 26 indicate mild, moderate, and severe stress, respectively. Higher scores reflect higher levels of these emotional states. The criteria for physical activity assessment are as follows: <19 = low; 20–42 = moderate; >43 = high level of physical activity.

**Table 3:** Dietary statistics.

Variable	N	Mean	Minimum	Maximum	Percentiles		
					*P*_25_	*P*_50_	*P*_75_
Cereals	683	2.27	‐12	12	‐4	5	8
Vegetables and fruits	683	‐7.26	‐12	‐1	‐10	‐8	‐4
Dairy products and soy	683	‐8.26	‐12	0	‐11	‐10	‐5
Animal-based foods	683	‐2.86	‐12	8	‐7	‐3	2
Pure energy foods	683	5.49	0	12	3	6	9
Condiments	683	2.50	0	5	0	3	4
Food variety	683	‐7.03	‐12	‐1	‐10	‐7	‐4
Diet quality distance	683	42.69	18	68	37	42	49

*Notes*: a score of 0 indicates that the diet has neither insufficient intake nor excessive intake. For diet quality distance (DQD), a score of 1 to 19 indicates relatively appropriate dietary balance, 20 to 38 indicates mild imbalance, 39 to 57 indicates moderate imbalance, and > 57 indicates severe imbalance.

### 3.3. Differences by grade

The results of the Kruskal–Wallis tests indicate significant differences in stress, anxiety, and depression levels (as well as overall mental health) between grade levels (all *P* < .05). Students in higher grades experienced substantially more psychological distress and reported worse mental health levels. Notably, however, no significant differences between grades were identified in physical activity or dietary quality, though there was a tendency for physical activity to decline across grades (see Table 4).

**Table 4:** Differences by grade.

Variable	Grade	N	Mean rank	Percentiles			*H*	*P*
				*P*_25_	*P*_50_	*P*_75_		
Stress	Freshman	235	291.54	10	12	14	34.965	<.001
	Sophomore	202	339.87	10	14	18		
	Junior	166	376.06	12	14	20		
	Senior	80	424.92	12	16	23.5		
Anxiety	Freshman	235	316.95	8	12	16	9.196	.027
	Sophomore	202	338.81	10	12	18		
	Junior	166	360.85	10	12	20		
	Senior	80	384.54	10	14	23.5		
Depression	Freshman	235	314.32	6	10	12	8.822	.032
	Sophomore	202	345.70	8	10	16		
	Junior	166	367.09	8	10	18		
	Senior	80	409.83	8	12	22		
Overall mental health	Freshman	235	301.02	28	32	38	22.359	<.001
	Sophomore	202	342.19	28	34	48		
	Junior	166	367.09	29.5	36	58		
	Senior	80	409.83	32	38	70		
Physical activity	Freshman	235	362.74	9	9	16	7.354	.061
	Sophomore	202	348.88	6	9	20		
	Junior	166	313.19	6	9	18		
	Senior	80	307.04	4	9	18		
Diet quality distance	Freshman	235	364.00	38	43	50	5.564	.135
	Sophomore	202	339.54	36	42	49		
	Junior	166	327.99	35	42	48		
	Senior	80	312.66	37	40.5	48.5		

### 3.4. Differences by gender

The results of the Mann–Whitney *U* tests indicate that females reported significantly higher stress, anxiety, and depression scores and lower overall mental health scores than males (all *P* < .05). Females also exhibited significantly lower physical activity levels (*P* = .013). No gender-based differences were identified when it came to dietary quality (see Table 5).

**Table 5:** Gender differences.

Variable	Grade	N	Mean rank	Percentiles			*Z*	*P*
				*P*_25_	*P*_50_	*P*_75_		
Stress	Male	261	318.99	10	12	16	‐2.414	.016
	Female	422	356.23	10	14	20		
Anxiety	Male	261	320.6	10	12	16	‐2.244	.025
	Female	422	355.24	10	12	18		
Depression	Male	261	322.79	6	10	14	‐2.013	.044
	Female	422	353.88	8	10	18		
Overall mental health	Male	261	321.69	28	34	42	‐2.119	.034
	Female	422	354.56	28	36	54.5		
Physical activity	Male	261	365.49	8	9	20	‐2.47	.013
	Female	422	327.47	6	9	16		
Diet quality distance	Male	261	349.35	37	43	49	‐0.766	.444
	Female	422	337.45	36	42	49		

### 3.5. Correlation analysis

Pearson correlation analysis revealed a negative relationship between mental health scores and physical activity (*r* = –0.307, *P* < .01), indicating that higher physical activity is linked to better mental health outcomes. Mental health scores were also positively associated with dietary quality distance (*R* = 0.106, *P* < .01), suggesting that higher dietary imbalances are related to worse mental health outcomes. No significant relationship was uncovered between physical activity and dietary quality (see Table 6).

**Table 6:** Correlation analysis.

Variable	Overall mental health	Physical activity	Diet quality distance
Overall mental health	1	‐.307*	.106*
Physical activity		1	‐0.022
Diet quality distance			1

* Indicates statistical significance at *P* < .05.

### 3.6. Intervention effects

Among the 440 sampled participants, the baseline demographics did not differ significantly between the randomized intervention group (n = 220) and control group (n = 220). Following the intervention, the intervention group exhibited significant improvements in stress, anxiety, depression, overall mental health, physical activity, and dietary quality, contrasting with the control group (all *P *< .05). More specifically, 44.5% of the participants in the intervention group exhibited improvements in mental health, 70.9% exhibited increased physical activity, and 84.1% exhibited an improved dietary balance, whereas the control group exhibited minimal change (see Tables 7 and 8).

**Table 7:** Pre–post comparison of intervention and control groups.

Variable	Group	Mean rank (pre)	Mean rank (post)	Median Q2 (Q1, Q3) pre	Median Q2 (Q1, Q3) post	*Z*	*P*
Stress	Intervention	227.2	189.74	14 (12, 20)	12 (10, 14)	‐10.542	<.001
	Control	213.8	251.26	14 (10, 22)	14 (10, 22)	‐1.732	.083
	*Z*	‐1.113	‐5.123				
	*P*	.266	<.001				
Anxiety	Intervention	214.11	194.24	13 (10, 20)	12 (10, 14)	‐7.820	<.001
	Control	226.89	246.76	14 (10, 20)	14 (10, 20)	‐1.000	.317
	*Z*	‐1.061	‐4.378				
	*P*	.289	<.001				
Depression	Intervention	225.22	205.05	12 (8, 16)	10 (8, 14)	‐8.499	<.001
	Control	215.78	235.95	10 (8, 20)	10 (8, 20)	‐.577	.564
	Z	‐0.784	‐2.569				
	*P*	.433	.010				
Overall mental health	Intervention	224.77	193.82	36 (32, 54)	32 (28, 41.5)	‐10.251	<.001
	Control	216.23	247.18	36 (30, 66)	36 (30, 66)	‐1.134	.257
	Z	‐0.706	‐4.413				
	*P*	.48	<.001				
Physical activity	Intervention	216.02	291.68	9 (6, 16)	25 (16, 45)	‐11.765	<.001
	Control	224.98	149.33	9 (6, 18)	9 (6, 17.5)	‐1.284	.199
	Z	‐0.747	‐11.786				
	*P*	.455	<.001				
Diet quality distance	Intervention	222.68	114.76	46 (42, 51)	29 (26, 33)	‐12.870	<.001
	Control	218.32	326.24	46 (41, 52)	45 (41, 51)	‐4.541	<.001
	Z	‐0.361	‐17.452				
	*P*	.718	<.001				

**Table 8:** Magnitude of change by group.

Group	Total (n)	Improved mental health	Improved physical activity	Improved diet quality
Intervention	220	98	156	185
Control	220	5	6	26
Improvement (%)		44.5%	70.9%	84.1%
		2.3%	2.7%	11.8%

## 4. Discussion

The study has demonstrated that university students in China are experiencing significant psychological distress, as evidenced by the mean scores for stress (*M* = 15.06), anxiety (*M* = 14.13), and depression (*M* = 12.71). These findings align with those of prior studies that have documented psychological disorders among students in various countries.^[16–18]^ This high prevalence coincides with the data from Auerbach et al,^[19]^ who, using data from WHO World Mental Health Surveys, documented the prominence of various mental disorders among international college students. It also aligns with their assertion that students in higher grades are more likely to exhibit higher stress, anxiety, and depression levels, making seniors the most vulnerable group among college students. This is likely because seniors are worrying about more advanced academic requirements as well as their future career and employability outcomes.

There were also some gender-based differences: Female students were found to be more stressed, anxious, and depressed than their male counterparts, aligning with the findings of other studies that have found female college students to be more prone to internalizing disorders.^[16,17]^ These results demonstrate that there is a need to address and tackle the gender gap in mental health support available to college students.

This study has also revealed a low average physical activity score (*M* = 11.98), with about 75% of students scoring even below the lowest activity level. More importantly, mental health scores were negatively correlated with physical activity (*r* = ‐0.307, *P* < .01), suggesting that physical activity is beneficial for mental health. This is consistent with a large body of work showing that physical activity reduces depression levels and improves mental health.^[20–23]^ This study’s intervention further confirmed these benefits: students in the intervention group reported greater increases in their physical activity and, correspondingly, greater improvements in their stress, anxiety, depression, and overall mental health levels. The evidence strengthens the case for physical activity as an effective, low-resource means of reducing and preventing depression symptoms.

The results also indicate a significant imbalance in the participants’ diets. The mean quality distance score of 42.69 signaled greater imbalance on a sample-wide scale. Students excessively and inappropriately filled their diets with grains, energy-dense foods, and condiments while insufficiently consuming vegetables, fruits, dairy, soy, and animal proteins. Mental health scores maintained a significant albeit weak positive correlation with dietary imbalance (*R* = 0.106, *P* < .01), suggesting poor dietary quality as a potential driver of mental health issues. This supports the increasingly apparent pattern of diet influencing the likelihood of depression.^[24–26]^ Some research has sought to mechanistically explain these relationships with a focus on the link between nutrient deficiency, inflammation, and gut dysbiosis.^[27]^ Other studies indicate that changes in dietary patterns, such as those undertaken in the SMILES trial, can mitigate depression.^[28]^

The intervention carried out in this study, which had the greatest impact on those in the intervention group when it came to dietary balance (84%), was evidently successful in promoting self-reported dietary quality. This adds to the body of literature, both observational and interventional, indicating that protective diet changes can alleviate depression symptoms and improve overall mental health.^[26,28]^

The intervention, which entailed lessons on mental health, the promotion of physical activity, and nutrition guidance, gave way to favorable outcomes across all considered metrics. The participants in the intervention program exhibited reduced stress, anxiety, and depression levels as well as higher levels of physical activity and improved eating habits, whereas those in the control group exhibited minimal change. These results constitute evidence that improved physical activity and nutrition can have additive positive impacts on mental health outcomes.^[23,27]^ The 44.5% improvement in mental health and 70.9% improvement in physical activity demonstrate the effectiveness of health-promotion programs that feature multiple components.

These observations should compel universities to implement organized, integrated programs aimed at improving mental health outcomes among their students. These programs should focus on promoting physical activity and nutrition in addition to providing psycho-social support. Refined strategies are also important in such programs: older students need stress relief to cope with the demands of their classes, while female students need targeted, sex-specific supportive health services. The incorporation of dietary counseling and physical activity programs in campus settings is likely to decrease stress, anxiety, and depression symptoms while enhancing students’ resilience.

Of course, even with this study’s strong results, its ability to establish causation was constrained by the cross-sectional design of the baseline data. While beneficial effects were noted, the intervention revealed that longer-term follow-ups are necessary to gauge sustainability. Self-reported data on diet and physical activity are also subject to potential recall bias. Subsequent studies should incorporate longitudinal evaluation and robust lifestyle correlates to determine causality in these relationships, particularly through evidence from biobehavioral factors.

## 5. Conclusion

This study has illustrated the complex interconnections of mental well-being, exercise, and dietary moderation among students in Guangdong, China. The results point to severe dietary imbalances and a lack of exercise, which are correlated with symptoms of stress, anxiety, and depression. Notably, the intervention exhibited significant effectiveness, bringing down stress, anxiety, and depression levels and improving physical activity, dietary intake, and, of course, overall mental health. These findings support the importance of promoting health through well-designed and evidence-informed initiatives to improve students’ mental and physical health. Such initiatives would broadly reduce mental health issues and promote positive behavioral changes among students across a wide range of academic environments.

## Acknowledgments

We sincerely thank the journal editors and anonymous reviewers for their diligent work and constructive comments; and Macau University of Science and Technology and the Hong Kong Public Health Technology Research Center for their continuous support in research environment and resources.

## Author contributions

**Data curation:** Jia Fu, Xuelan Wu.

**Formal analysis:** Xuelan Wu.

**Investigation:** Jia Fu, Xuelan Wu.

**Methodology:** Jia Fu, Xuelan Wu, Tian Zhong.

**Project administration:** Jia Fu.

**Resources:** Xi Yu, Tian Zhong.

**Software:** Xuelan Wu, Xi Yu, Tian Zhong.

**Supervision:** Ying Xiao, Xiaoyu Tao.

**Writing – original draft:** Jia Fu.

**Writing – review & editing:** Jia Fu, Ying Xiao, Xiaoyu Tao.
